# 139 Physical literacy in adolescents with disabilities in Ireland: a cross-sectional study examining effects of disability, age, gender and family affluence on physical literacy

**DOI:** 10.1093/eurpub/ckae114.099

**Published:** 2024-09-26

**Authors:** Una Britton, Kwok Ng, Stephen Behan, Hannah Goss, Paul McFlynn, Julia McClelland, Catherine Woods, Sinead Connolly, Sarahjane Belton

**Affiliations:** Dublin City University, Dublin, Ireland

## Abstract

**Purpose:**

Physical literacy (PL), defined as physical competence, confidence, motivation, and knowledge and understanding, is the foundation for physical activity (PA). PL is inclusive of all, yet PL research has proved quite exclusive, with a scarcity of data on those with disabilities. In Ireland, National PA Report Cards present contrasting grades for those with and without disabilities, suggesting different PA experiences which impact PL. Factors consistently recognised as correlates of PA in the general population (sex, age, socioeconomic status), and how they impact PL, are not well understood in the disability population. This research reports on PL in adolescents with disabilities attending mainstream education, investigating differences between those with and without disabilities.

**Methods:**

Participants were adolescents involved in the Children’s Sport Participation and Physical Activity study (Woods et al 2023. Age, gender, family affluence and disability status were recorded. Perceived physical competence (McGrane et al 2015), PA self-efficacy (Marcus et al 1992), motivation (14 items), and PA knowledge and understanding (4 questions) were measured. Difference in PL by disability status was analysed using one-way MANOVA and Chi-square tests for independence. Three-way MANOVA investigated effects of age, gender, and family affluence on PL.

**Results:**

Participants were 3,964 adolescents (Mage 14.9±1.87 years). Perceived physical competence, PA self-efficacy and PA knowledge and understanding were significantly lower amongst those with disabilities (small effect sizes; Ƞ2 = 0.053, 0.027, 0.021 respectively). Significantly more children with disabilities cited “something to do” and “because of mother” as motivating factors. Those with motoric disabilities had significantly poorer PL than other disability types. Being female and/or of low family affluence negatively impacted PL. Gender had a greater effect on PL in those without disabilities (Ƞ2 = .040-.044) than with (Ƞ2 = .027-.032). Family affluence had a greater effect on PL in those with disabilities (Ƞ2 = .028-.032) than without (Ƞ2 = .017-.023).

**Conclusion:**

Having a disability puts children at risk of poor PL, with family affluence having a greater effect on PL for those with disabilities. PA policies should focus on improving PL in children with disabilities, particularly those from low family affluence.

**Funding:**

Sport Ireland/Northern Ireland, Healthy Ireland.

